# Development of an efficient one-step real-time reverse transcription polymerase chain reaction method for severe acute respiratory syndrome-coronavirus-2 detection

**DOI:** 10.1371/journal.pone.0252789

**Published:** 2021-06-04

**Authors:** Yukiko Nakura, Heng Ning Wu, Yuya Okamoto, Muneyuki Takeuchi, Koichiro Suzuki, Yoshitaka Tamura, Yuichiro Oba, Fumiko Nishiumi, Nobuaki Hatori, Shinsuke Fujiwara, Kiyoshi Yasukawa, Shinobu Ida, Itaru Yanagihara

**Affiliations:** 1 Department of Developmental Medicine, Research Institute, Osaka Women’s and Children’s Hospital, Izumi-city, Osaka, Japan; 2 Department of Laboratory Medicine, Osaka Women’s and Children’s Hospital, Izumi-city, Osaka, Japan; 3 Department of Intensive Care Medicine, Osaka Women’s and Children’s Hospital, Izumi-city, Osaka, Japan; 4 The Research Foundation for Microbial Diseases of Osaka University, Suita-city, Osaka, Japan; 5 Department of Clinical Laboratory, Osaka Habikino Medical Center, Habikino-city, Osaka, Japan; 6 Department of General Medicine, Osaka General Medical Center, Osaka-city, Osaka, Japan; 7 Department of Biosciences, School of Biological and Environmental Sciences, Kwansei-Gakuin University, Sanda-city, Hyogo, Japan; 8 Division of Food Science and Biotechnology, Graduate School of Agriculture, Kyoto University, Kyoto-city, Kyoto, Japan; Waseda University: Waseda Daigaku, JAPAN

## Abstract

The general methods to detect the RNA of severe acute respiratory syndrome-coronavirus 2 (SARS-CoV-2) in clinical diagnostic testing involve reverse transcriptases and thermostable DNA polymerases. In this study, we compared the detection of SARS-CoV-2 by a one-step real-time RT-PCR method using a heat-resistant reverse transcriptase variant MM4 from Moloney murine leukemia virus, two thermostable DNA polymerase variants with reverse transcriptase activity from *Thermotoga petrophila* K4 and *Thermococcus kodakarensis* KOD1, or a wild-type DNA polymerase from *Thermus thermophilus* M1. The highest performance was achieved by combining MM4 with the thermostable DNA polymerase from *T*. *thermophilus* M1. These enzymes efficiently amplified specific RNA using uracil-DNA glycosylase (UNG) to remove contamination and human *RNase P* RNA amplification as an internal control. The standard curve was obtained from 5 to 10^5^ copies of synthetic RNA. The one-step real-time RT-PCR method’s sensitivity and specificity were 99.44% and 100%, respectively (n = 213), compared to those of a commercially available diagnostic kit. Therefore, our method will be useful for the accurate detection and quantification of SARS-CoV-2.

## Introduction

The COVID-19 pandemic, caused by the RNA virus SARS-CoV-2, has wreaked havoc on the global economy and many national healthcare systems. Countries with a large number of daily new cases need a reliable and inexpensive diagnostic system. In other countries, there is also an urgent need to stockpile kits for emergency use.

Currently, nucleic acid detection and antigen detection are the two primary methods to confirm infection. Real-time quantitative PCR (RT-qPCR) is a highly sensitive and widely used method for detecting SARS-CoV-2 RNA. Fully automated platforms for RT-qPCR have been introduced in large medical institutions and clinical laboratories. However, there is still a global shortage of reagents and consumables. In addition, RT-qPCR involves complicated molecular biological techniques. Moreover, RT-qPCR produces false-positive [1] or false-negative results [2].

The conventional RT-PCR consists of two steps, the synthesis of cDNA using reverse transcriptase and the amplification of DNA using DNA polymerase. In contrast, one-step real-time RT-PCR is a continuous reaction that performs the two procedures in the same tube. Therefore, it is essential to adjust the reaction buffer conditions so that two or more enzymes remain functional in the same buffer.

In this study, we applied the widely used primers and probes, reported by the United States Centers for Disease Control and Prevention (CDC), in RT-PCR to detect the SARS-CoV-2 RNA. Previously, we reported a thermostable reverse transcriptase, MM4, harboring 4 amino acid substitutions in the original Molony murine leukemia virus reverse transcriptase [3]. In addition, we selected 2 available family A DNA polymerase, DNA polymerase from *Thermus thermophilus* M1 strain (M1pol_Tth_) with a 5ʹ–3ʹ but not a 3ʹ–5ʹ exonuclease domain [4], and the genetically engineered L329A DNA polymerase variant (K4pol_L329A_) originated from the *Thermotoga petrophila* K4 strain. K4pol_L329A_ has acquired reverse transcriptase activity via mutagenesis; it has a 3ʹ–5ʹ but not a 5ʹ–3ʹ exonuclease domain [5]. RTX is xenopolymerase harboring reverse transcriptase and DNA polymerase activities due to the introduction of 17 amino acid substitutions [6] in a Family B KOD DNA polymerase from *Thermococcus kodakarensis*. Lastly, we used family I uracil-DNA glycosylase (UNG), cleaving the N-glycosylic bond between uracil and sugar to remove the uracil incorporated in DNA, to remove contaminations from the PCR products [7].

Currently, the clinical detection range of the RNA copy number for SARS-CoV-2 varies. The one-step real-time RT-PCR that we developed was evaluated for its detection range compared to that of a commercial SARS-CoV-2 detection kit authorized by the Ministry of Health, Labour and Welfare, Japan, as in vitro diagnostics (https://www.mhlw.go.jp/stf/newpage_11332.html). We aimed to detect 10–10^5^ copies of RNA per test within 45 PCR cycles.

Eventually, we successfully amplified 5–10^5^ copies of synthetic SARS-CoV-2 RNA and confirmed the specific amplification of the viral RNA from clinically isolated RNA samples.

## Materials and methods

### Purification of MM4 reverse transcriptase, M1pol_Tth_, K4pol_L329A_, and RTX DNA Polymerase

Recombinant MM4 [3], M1pol_Tth_ [4], K4pol_L329A_ [5], and RTX [6, 8] were purified, as previously described. Protein concentrations were measured with a NanoDrop 2000 (Thermo Fischer Scientific, Waltham, MA, USA). Then, the protein molar concentrations were calculated.

### RNA samples and RT-qPCR

The clinical samples were obtained from Osaka Women’s and Children’s Hospital, Osaka Habikino Medical Center, and Osaka General Medical Center of the Osaka Prefectural Hospital Organization. The ethics committee of Osaka Women’s and Children’s Hospital (Approval number 1365, 1365–2, 1365–3) approved this study for emergent clinical research. Samples were obtained according to the active epidemiological investigation requested from the Ministry of Health, Labour and Welfare, Japan, under the Japanese law of Act on the Prevention of Infectious Diseases and Medical Care for Patients with Infectious Diseases (the Infectious Diseases Control Law). And the residual RNA was utilized for this study after opting out.

RNA was extracted from a total of 213 samples, including at least 35 sputum samples, 124 nasopharyngeal swabs, and 7 saliva samples, using the QIAamp Viral RNA Mini Kit (QIAGEN, Valencia, CA, US). Uracil-DNA glycosylase (UNG), RNase inhibitor, and deoxyribonucleotides (a mixture of A, C, G, and U) were purchased from Toyobo Co., Ltd., Osaka, Japan. One-step real-time RT-PCR was performed using the QuantStudio 5 Real-time PCR system (Thermo Fisher Scientific). RNA copy numbers were estimated from the simultaneously tested standard curve.

Primer and probes for 2019-Novel Coronavirus (2019-nCoV) were synthesized according to the report from CDC (https://www.cdc.gov/coronavirus/2019-ncov/lab/rt-pcr-panel-primer-probes.html), including the 2019-nCoV_N1 Forward Primer (5ʹ-GACCCCAAAATCAGCGAAAT-3ʹ), 2019-nCoV_N1 Reverse Primer (5ʹ-TCTGGTTACTGCCAGTTGAATCTG-3ʹ), 2019-nCoV_N1 probe (5ʹ-FAM- ACCCCGCATTACGTTTGGTGGACC-TAMRA-3ʹ), 2019-nCoV_N2 Forward Primer (5ʹ-TTACAAACATTGGCCGCAAA-3ʹ), 2019-nCoV_N2 Reverse Primer (5ʹ-GCGCGACATTCCGAAGAA-3ʹ), 2019-nCoV_N2 probe (5ʹ-FAM-ACAATTTGCCCCCAGCGCTTCAG-TAMRA-3ʹ), RNase P forward primer (5ʹ-AGATTTGGACCTGCGAGCG-3ʹ), and RNase P Reverse primer (5ʹ- GAGCGGCTGTCTCCACAAGT-3ʹ). RNase P probe was adjusted from the CDC probe for minor groove binder (MGB) coupled with non fluorescence quencher: MoCO RNase P probe 5ʹ-VIC-TTCTGACCTGAAGGCT-MGB-3ʹ (Thermo Fisher Scientific). The synthetic standard RNA for the COVID-19 CDC N1 and N2 primer sets (US-CDC-N1N2-PC) (Nihon Gene Research Laboratories Inc., Sendai, Japan) was purchased. The synthetic standard RNA at the concentration of 10^7^ copies /μL was then serially diluted to 10^5^, 10^4^, 10^3^, 10^2^, 10, and 5 copies/μL in RNA grade TE buffer (Nacalai Tesque, Inc., Kyoto, Japan). The results from our one-step real-time RT-PCR data were compared with those obtained from a commercial one-step RT-PCR kit (SARS-CoV-2 direct PCR detection kit, Takara Bio Inc., Kusatsu, Japan).

### Statistical analysis

The data were analyzed by one-way ANOVA. The difference among the mean Ct values was analyzed using the Tukey–Kramer test. Sensitivity, specificity, positive predictive value (PPV), negative predictive value (NPV), and bivariate relationship were analyzed using the JMP 10 software (SAS) or Igor Pro (WaveMetrics).

## Results and discussion

### Buffer optimization for one-step real-time RT-PCR using a fluorescent probe

Five buffers with different concentrations of MgCl_2_, Mn(OCOCH_3_)_2_, Bicine-KOH (pH 8.2), Tris-HCl (pH 8.3), KCl, and CH_3_COOK supplemented with purified total human RNA were prepared (Table 1). Mn^2+^, required for the reverse transcriptase activity of *Tth* DNA polymerase [9], was included in RT buffers 1, 2, and 5.

**Table 1:** The composition of five different 5 × buffers.

Buffer	1	2	3	4	5
MgCl_2_ (mM)	15	7.5	7.5	7.5	5
Mn(OCOCH_3_)_2_ (mM)	5	6.5	0	0	5
Bicine-KOH, pH 8.2 (mM)	250	325	0	325	250
Tris-HCl, pH 8.3 (mM)	100	162.5	50	162.5	100
KCl (mM)	50	325	250	325	50
CH_**3**_COOK (mM)	500	195	0	195	500
tRNA μg /mL	65	65	65	65	65

**Fig 1:**
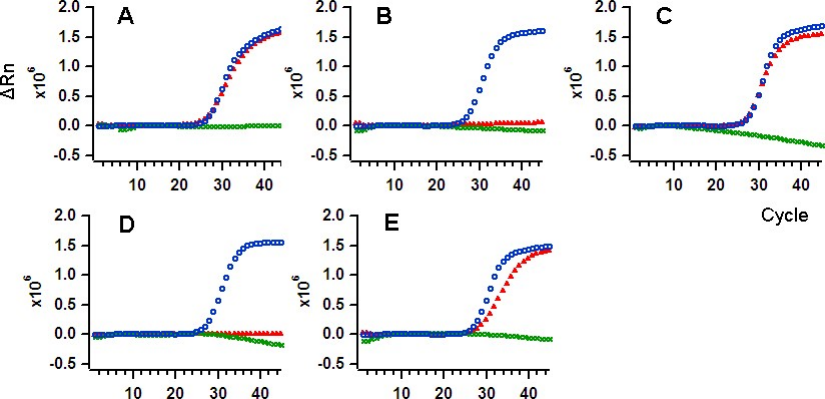
The amplification plot of various sample buffers with DNA polymerases. The amplification plots were obtained using MM4 in combination with K4pol_L329A_ (red, triangle), RTX (green, cross), or M1pol_Tth_ (blue, open circle). Here, 10^4^ copies of synthetic RNA were used as the template. The sample buffers used were as follows, A. Buffer 1, B. Buffer 2, C. Buffer 3, D. Buffer 4, and E. Buffer 5.

One-Step real-time RT-PCR was performed by mixing MM4 with the respective DNA polymerases, M1pol_Tth_, K4pol_L329A_, and RTX to verify 10^4^ and 10^3^ copies of SARS-CoV-2 RNA. We found that M1pol_Tth_ enabled the detection of specific RNA in all the buffers, while K4pol_L329A_ could amplify the target sequence only in buffers 1, 3, and 5. The family B DNA polymerase, RTX, lost its amplification ability with a fluorescent probe. On the other hand, M1pol_Tth_ combined with MM4 achieved amplification in various buffers (Fig 1). While we analyzed clinical specimens, we found that M1polTth, less affected by buffer compositions, was likely the most suitable DNA polymerase for one-step real-time RT-PCR. There was variation in the Ct values of the amplification of 10^3^ or 10^4^ copies of RNA (Table 2) by different enzymes in multiple buffers. When M1pol_Tth_ DNA polymerase was used, there was no significant difference in the sensitivity (Ct value) in detecting 10^4^ or 10^3^ copies of RNA per test.

**Table 2:** The Ct value of the one-step real-time PCR with MM4 and K4pol_L329A_, RTX, or M1pol_Tth_.

	K4pol_L329A_		RTX		M1pol_Tth_	
Standard RNA (copies)	10^4^	10^3^	10^4^	10^3^	10^4^	10^3^
Buffer 1	25.93^a^	30.20	nd^b^	nd	25.36	29.08
Buffer 2	nd	nd	nd	nd	25.47	29.23
Buffer 3	26.40	29.87	nd	nd	26.30	29.87
Buffer 4	nd	nd	nd	nd	25.60	29.19
Buffer 5	27.56	30.83	nd	nd	25.79	29.46

^a^Mean Ct value; n = 2.

^b^nd, not detected.

**Fig 2:**
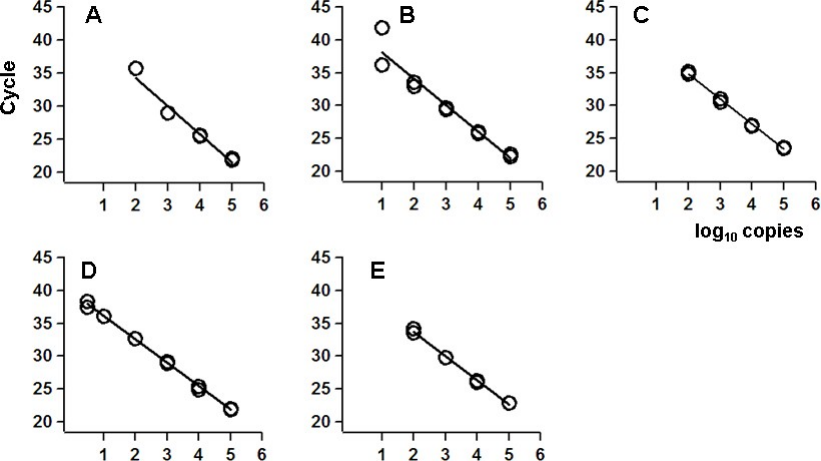
Analysis of the detection limit with each buffer. The amplification plot of each buffer with MM4 andM1pol_Tth_ was obtained. Standard synthetic RNA was used as the template at 10^5^, 10^4^, 10^3^, 10^2^, 10, and 5 copies per reaction. The sample buffers used were as follows, A. Buffer 1, B. Buffer 2, C. Buffer 3, D. Buffer 4, and E. Buffer 5.

Next, the detection limits of the method in each buffer with MM4 and M1pol_Tth_ were compared. In buffers 1, 3, and 5, the one-step real-time RT-PCR system could detect more than 100 copies of RNA. On the other hand, the method could detect more than 10 copies of RNA in buffer 2 and more than 5 copies in buffer 4 (Fig 2 and Table 3). Each Ct value obtained in buffer 4 was smaller than those in the other buffers. Therefore, buffer 4, combined with M1pol_Tth_, exhibited the best performance in the tested conditions.

**Table 3:** The Ct values of the one-step real-time PCR with MM4 and M1pol_Tth_ with standard RNA of varying copy numbers.

	Buffer				
Standard RNA (copies)	1	2	3	4	5
100,000	22.00^a^	22.44	23.59	21.61	22.91
10,000	25.57	25.95	27.01	24.82	26.11
1,000	29.02	29.54	30.81	28.28	29.80
100	35.80	33.28	35.00	32.09	33.88
10	nd^b^	39.03	nd	35.41	nd
5	nd	nd	nd	36.54	nd

^a^Mean Ct value; n = 2.

^b^nd, not detected.

We further optimized the reaction time of the reverse transcriptases. MM4’s reaction time was set from 30 sec to 15 min at 50°C to test the detection of 10, 10^2^, and 10^4^ copies of synthetic RNA. The Ct value did not vary significantly when the initial standard RNA copies were low (10 and 100 copies/test). On the other hand, Ct values at 30 sec were significantly higher than at 5, 10, and 15 min with 10^4^ RNA copies. Therefore, the optimal reverse transcription reaction time was determined to be 5 min (Table 4).

**Table 4:** The effect of RT incubation time and standard RNA copy numbers on the Ct values of the one-step RT-PCR with MM4 and M1pol_Tth_.

Standard RNA	RT incubation time (Mean Ct values ± S.D.)			
copy numbers	30 sec	5 min	10 min	15 min
10,000	25.60 ± 0.05*	25.02 ± 0.04	25.03 ± 0.18	25.02 ± 0.06
100	32.81 ± 0.15	32.30 ± 0.45	32.24 ± 0.16	32.43 ± 0.07
10	36.35 ± 0.27	35.46 ± 0.50	35.66 ± 0.90	36.13 ± 0.71

Mean Ct values ± S.D; n = 3.

*p < 0.05 (Tukey–Kramer HSD).

PCR contamination due to amplified DNA carryover is a problem in clinical testing. Therefore, we examined the applicability of UNG to our one-step real-time RT-PCR method. We found that the contamination of PCR-amplified DNA could be avoided by adding UNG at 0.4 U/test. On the other hand, the *RNase P* gene has been used as an internal standard in coronavirus detection systems. Thus, we investigated whether the CDC primer and probe sets could be used for the simultaneous detection of *RNase P*. We found that our method could be used as an internal standard. The final, optimized conditions and cycling parameters for the one-step real-time RT-PCR were determined (Tables 5 and 6). Our one-step real-time RT-PCR kit was designated as the “Mother’s and Children’s, Osaka” (MoCO) kit.

**Table 5:** Optimized RT-PCR conditions for SARS-CoV-2 detection with the MoCO kit.

Reagent	Volume (μL)
5 × RT Buffer 4	4
dNTPs Mixture (2 mM of A, C, G, and U)	1.5
M1pol_Tth_ (30 μM)	1
RNase Inhibitor, Recombinant	0.5
MM4 (2 μM)	1
RNase P primer, forward (10 μM)	0.1
RNase P primer, reverse (10 μM)	0.1
RNase P probe (5 μM)	0.08
2019-nCoV_ primer, forward (10 μM)	1
2019-nCoV_ primer, reverse (10 μM)	1
2019-nCoV_ probe (5 μM)	0.8
Uracil-DNA Glycosylase (UNG), Heat-Labile (1 unit/μL)	0.4
RNA (standard or sample RNA)	3
Double distilled water	Up to 20 μL

**Table 6:** Optimized one-step real-time RT-PCR cycling parameters for the MoCO kit.

Temperature	Duration	Number of cycles
25°C	10 min	1
50°C	5 min	1
95°C	30 sec	1
95°C	10 sec	45
60°C	30 sec	

### Consistency with the commercial kit with clinical RNA samples

**Fig 3:**
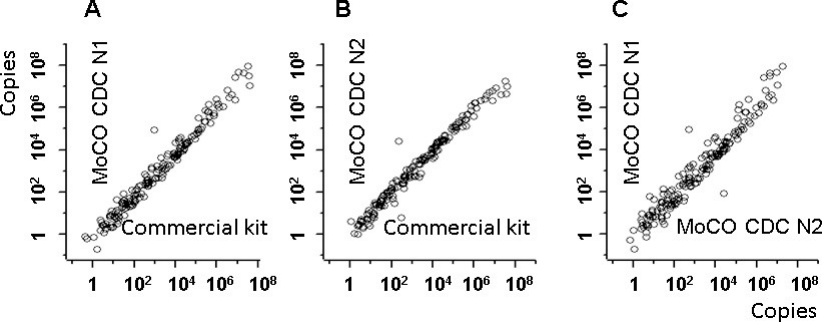
Log-log plot analysis of the MoCO kit compared to the commercial kit. A. The MoCO kit with the CDC N1 primers and probe set was compared to the Takara Kit with the CDC N1 and N2 primers and probes. B. The MoCO kit with the CDC N2 primers and probe set was compared to the Takara Kit with the CDC N1 and N2 primers and probes. C. The MoCO kit with the CDC N1 primers and probe set was compared to the MoCO kit with the CDC N2 primers and probe set (n = 213).

One-step real-time RT-PCR was performed with the MoCO kit and a commercial kit (SARS-CoV-2 direct PCR detection kit, Takara Bio Inc., Kusatsu, Japan) on a total of 213 RNA samples (S1 Table). Clinical RNA specimens were stored at -80°C before use. The MoCO kit detected using the N1 and N2 of the CDC probes separately, while both probes are mixed in the Takara kit. The MoCO kit’s accuracy was checked by setting the detection limits at 10, 5, and 1 copies; the sample was considered positive if amplification with the N1 or N2 probe exceeded the detection limit. With the detection limit of 10 copies, the MoCO kit’s sensitivity, specificity, PPV, and NPV were 98.1, 96.5, 98.7, and 94.8%, respectively. With the detection limit of 5 copies, its sensitivity specificity, PPV, and NPV were 98.2, 97.9, 99.4, and 94.0%, respectively. With the detection limit of 1 copy, the kit’s sensitivity, specificity, PPV, and NPV were 99.4, 100, 100, and 97.2%, respectively (Table 7). The log-log plots were well fitted in a wide range (Fig 3). These results indicate that the MoCO kit is clinically applicable for detecting SARS-CoV-2 RNA.

**Table 7:** Performance evaluation of the MoCO kit.

	Detection limit		
	Percentage (%) of samples with copies:		
	<10	<5	<1
Sensitivity	98.1	98.2	99.4
Specificity	96.5	97.9	100
PPV^a^	98.7	99.4	100
NPV^b^	94.8	94.0	97.2

^a^PPV, positive predictive value.

^b^NPV, negative predictive value.

Our developed MoCO kit could reproducibly detect at least 5 copies of synthetic RNA (Fig 2), demonstrating comparable performance to that of the commercial kit (Fig 3 and Table 7). One of the critical problems in the clinical amplification of high-sensitive nucleic acids is cross-contamination. The MoCO kit can be used in combination with UNG to minimize DNA carryover. In addition, by using a thermostable reverse transcriptase, MM4, the kit could achieve cDNA synthesis efficiently at 50°C for 5, 10, and 15 min (Table 4). Our protocol does not require pre-heating at 65°C during cDNA synthesis. Also, our method could simultaneously detect *RNase P* as an internal standard; the one-tenth of the amount of primers and probes for SARS-CoV-2 was sufficient for *RNase P*. Our highly sensitive RT-PCR method is expected to be applied to identifying other pathogens and analyzing mutations.

## Conclusions

The MoCO kit was developed to detect SARS-CoV-2 from the extracted RNA samples. Our development kit performed well for emergency research tests with a detection limit of 5 copies of RNA. It is necessary to continue improving the kit by simplifying sample preparation and the diagnosis of viral RNA mutations. Our high-sensitive RT-PCR method is expected to be applied for the molecular detection of various infectious diseases.

## Supporting information

S1 Table Comparison of the SARS-CoV-2 copy numbers detected using the MoCO kit and a commercial kit (SARS-CoV-2 direct PCR detection kit, Takara). (PDF)
