# Supplementary material for: Development of an efficient one-step real-time reverse transcription polymerase chain reaction method for severe acute respiratory syndrome-coronavirus-2 detection
Source: PLoS One. 2021 Jun 4;16(6):e0252789. doi: 10.1371/journal.pone.0252789 (PMC8177496; doi:10.1371/journal.pone.0252789)
Supplement: S1 Table — (PDF) [file pone.0252789.s001.pdf]

S1.Table

No.	MoCO N1	MoCO N2	TAKARA
	Quantity	Quantity	Quantity
1	81	27	29
2	46,337,472	8,934,109	11,692,693
3	2.3	4.8	9.8
4	603,690	280,460	357,953
5	721	248	332
6	25,738	13,038	14,263
7	14	8.4	14
8	90,576	506	927
9	320,642	113,385	133,275
10	4,162	2,069	1,981
11	43	10	14
12	127	70	78
13	112	63	112
14	4,858	9,613	10,893
15	1,695	538	1,203
16	1,401	595	687
17	2,112	788	2,617
18	93,276,416	18,398,274	31,407,314
19	2.0	-	8.6
20	-	-	-
21	-	-	-
22	6.7	8.8	6.6
23	6.9	6.3	12
24	838	1,028	1,236
25	51	44	61
26	469	511	2,088
27	125,370	183,626	184,490
28	77	148	139
29	6,081	12,658	10,011
30	65,009	126,141	109,819
31	2,017	4,252	4,104
32	0.6		0.5
33	-	-	-
34	1,893	5,593	5,732
35	31,633	118,986	87,343
36	541,068	1,242,466	721,372

37	-	-	-
38	16,657	23,594	31,359
39	222,072	400,665	274,133
40	21	26	31
41	283	584	421
42	68	111	99
43	0.2	1.1	1.6
44	93,351	214,100	171,440
45	4,361	6,422	9,094
46	293	357	364
47	11,914	24,352	16,131
48	1.2	4.2	3.4
49	9,137	15,630	14,523
50	3,324	4,708	11,612
51	467	524	1,477
52	4,754	12,433	9,820
53	2,307,317	6,261,374	7,957,190
54	6.8	24	19
55	178,126	474,751	492,082
56	4,612	11,088	9,290
57	6,676	10,627	9,055
58	6,568	13,791	13,234
59	1.8	2.3	3.9
60	25,225	28,354	45,302
61	28	37	30
62	-	-	-
63	13	14	8.3
64	1.5	1.0	2.4
65	32	25	28
66	984	2,092	2,421
67	5.9	8.3	13
68	11,463,843	9,922,947	38,618,664
69	29	36	31
70	4.9	2.4	2.4
71	274	501	479
72	1,825	3,239	3,165
73	2.6	4	3.2
74	6.8	12	11
75	2.7	5	3.5
76	39	51	33

77	239	253	379
78	0.7	4.1	1.1
79	26	98	72
80	36	270	111
81	25	108	90
82	55	297	176
83	4,200,647	5,090,626	5,534,097
84	407	1,242	801
85	1,157,088	2,143,965	2,004,790
86	693	3,275	2,410
87	1,211	4,667	3,523
88	7,867	22,216	18,041
89	19,574	44,953	36,742
90	2.9	10	7.6
91	404,562	748,434	723,929
92	10,792	32,220	25,581
93	5,914	15,199	13,364
94	422	1,449	1,051
95	50	150	95
96	27	73	39
97	1,151	2,701	1,422
98	8,994	23,896	22,482
99	455	1,706	1,069
100	7.8	41	33
101	256	934	736
102	182	604	469
103	3.1	4.1	4.0
104	5.5	24	14
105		1.9	3.9
106	23,822	31,480	39,559
107	3.0	31	15
108	8.0	5.3	7.2
109	23	71	76
110	11	26	17
111	366	489	428
112	11,253	27,438	25,536
113	58,112	75,010	105,040
114	4.2	25	17
115	195	612	436
116	6,866	5,360	8,457

117	37	90	60
118	43	129	104
119	25	47	41
120	4,546	7,279	8,348
121	1,699	4,032	4,647
122	32,894,664	4,409,194	34,734,640
123	270	567	409
124	32	119	98
125	1,182	3,498	2,939
126	5,718	11,843	11,966
127	45,472,364	4,283,486	21,012,826
128	-	-	0.6
129	86	25,381	213
130	276	1,134	541
131	40,204	9,577	17,285
132	27,239,298	2,153,792	7,932,101
133	1,408,114	135,687	391,362
134	756,957	122,457	286,134
135	568	151	398
136	2,558,073	389,504	929,264
137	20,939	3,973	9,806
138	34,149	8,885	14,763
139	6,327	3,293	4,163
140	16	22	34
141	196	170	198
142	7.6	5.0	6.5
143	211,571	256,941	248,158
144	2.7	2.8	2.7
145	12	8.5	13
146	8.5	5.9	14
147	14,998	20,108	19,720
148	7,028	6,802	8,878
149	403	350	416
150	76	47	114
151	3,043,654	3,814,454	4,092,303
152	208	244	354
153	87,243	121,275	124,910
154	993,015	962,130	1,280,356
155	3.7	2.6	2.6
156	9.0	2.5	9.1

157	10,885	22,905	17,846
158	19	19	25
159	95,933	161,568	127,282
160	1,515,474	1,706,045	1,178,881
161	47,938	96,394	79,002
162	393,905	469,028	434,257
163	33	23	43
164	20,500	49,271	38,292
165	185	352	175
166	32	24	25
167	1,266,018	1,671,644	1,046,217
168	1,362	2,272	2,163
169	39	42	30
170	70,860	89,477	128,593
171	20,100	30,097	30,437
172	36	101	59
173	21,356	29,061	33,206
174	0.04		2.0
175	-	-	0.5
176	40,857	38,935	63,514
177	-	-	-
178	17	63	40
179	2.1	3.1	7.0
180	21	61	65
181	38	160	108
182	136	29	138
183	628,327	284,529	422,225
184	6,544,307	2,556,935	3,286,751
185	5,125	3,103	3,842
186	60	6.2	284
187	1247	466	694
188	-	-	-
189	0.8	-	0.4
190	0.5	0.7	-
191	-	-	-
192	-	-	-
193	-	-	-
194	-	-	-
195	-	-	-
196	-	-	-

197	-	-	-
198	-	-	-
199	-	-	-
200	-	-	-
201	-	-	-
202	-	-	-
203	-	-	-
204	-	-	-
205	-	-	-
206	-	-	-
207	-	-	-
208	-	-	-
209	-	-	-
210	-	-	0.4
211	-	-	-
212	-	-	-
213	-	-	-
